# Editorial: Immunesenescence in innate immune cells and disease development

**DOI:** 10.3389/fimmu.2025.1563373

**Published:** 2025-02-13

**Authors:** Rania Hassan Mohamed

**Affiliations:** Department of Biochemistry, Faculty of Science, Ain Shams University, Cairo, Egypt

**Keywords:** aging, immunesenescence, immune-associated diseases, macrophages, immune exhaustion

Population ageing is accelerating worldwide. As per the WHO, the world’s population over 60 years will double by 2050 with 80% living in low and middle income countries. The elderly are highly susceptible to complex age-related-diseases, which represent more economic burden on the medical care systems. Thus, the UN has announced (2020-2030) as the decade of healthy aging in alignment with the last ten years of the SDGs. Despite the presence of different therapeutic strategies, many diseases are spreading in the elderly and are accompanied by therapy resistance. As the age progresses, the immune system losses its proper functional balance, protection against pathogens and homeostatic properties in a condition called inflammaging. The inflammaging is characterized by chronic low-grade inflammation and tissue damage development, which leads to immunesenescence defined as the decline in immune cell function. Moreover, there is an ongoing wave of research supporting the therapeutic role of lifestyle and pharmacological interventions in rejuvenating aged immune system to promote healthy ageing. In this Research Topic, we have compiled research articles contributing to our understanding of disruption of immune system related to immunesenescence and associated diseases.

Soraci et al. addressed the updates and recent advances in innate immunesenescence features related to neurocryptococcosis infection. Cryptococcus neoformans (C. Neoformans andC. gattii) infectious disease of central nervous system (CNS) increases in older adults. The authors demonstrated the immunesenscence as the putative cause of the increased susceptibility to infections in older people. This increase incidence of infection was attributed to the enhanced pro-inflammatory activity of macrophage M1 cells, increased production of Th2 cells and their cytokines, impaired inflammatory response of neutrophils and increased IFN-I mediated neuroinflammatory status related to inflammaging. Dissecting these underlying molecular mechanisms of immunesenescence impact on neurocryptococcosis infection improves understanding of new approaches for prevention and recognition of neurocryptococcosis in older adults.

Immune exhaustion shares overlapping features with immunesenescence; Surendar et al. showed a unique signature of the immune exhaustion in osteomyelitis (OMS). By the detailed enumeration of innate and adaptive immune cells, and their markers, they found increased anti-inflammatory pattern. This pattern was evidenced by increased immune exhaustion phenotype and immune regulatory cells, in addition to decreased immune activation markers along with pronounced involvement of the Tfh memory B cell axis. Together, the study suggested immune modulating therapy as a promising alternative against the OMS infection to dampen the microbial evasion mechanisms in the current era of increased antibiotic resistance.

Other diseases associated with immune features that are common in immunesenescence are also addressed in this Research Topic such as, non-alcoholic fatty liver disease (NAFLD) and Aspergillus fumigatus keratitis. The elderly usually suffer from the NAFLD complications more than the young people. Guo et al. reviewed the advances of the cutting-edge technologies such as, single-cell RNA sequencing (scRNA seq) and time-of-flight mass spectrometry flow cytometry, etc … in identification of the innate and adaptive immunological mechanisms involved in the NAFLD pathology. They improved the understanding of the accumulation and activation molecular mechanisms of immune cells in NAFLD. Senescent microenvironment is characterized by accumulation of innate immune cells, especially macrophage. Guibo et al. studied the molecular mechanisms driving the polarization of macrophages in Aspergillus fumigatus infection. The studypresented the Dectin-1 as an indispensable player in inflammatory macrophage polarization in the antifungal innate immunity through the MAPK signaling pathway in fungal keratitis.

Highlighting the mechanisms involved in immunesenescence will improve the understanding of the development, interpretation, diagnosis or manipulation of its associated diseases. The four articles published in this Research Topic have contributed to address the updates and recent advances in immunesenescence microenvironment. We believe the outcomes of these research groups also suggested potential markers and therapeutic targets combatting immunesenscence.

